# Downregulation of Anger by Mental Contrasting With Implementation Intentions (MCII)

**DOI:** 10.3389/fpsyg.2018.01838

**Published:** 2018-10-04

**Authors:** Inge Schweiger Gallo, Maik Bieleke, Miguel A. Alonso, Peter M. Gollwitzer, Gabriele Oettingen

**Affiliations:** ^1^Departamento de Antropología Social y Psicología Social, Facultad de Ciencias Políticas y Sociología, Complutense University of Madrid, Campus de Somosaguas, Madrid, Spain; ^2^Department of Psychology and Graduate School of Decision Sciences, University of Konstanz, Konstanz, Germany; ^3^Departamento de Psicología Social, del Trabajo y Diferencial, Facultad de Psicología, Complutense University of Madrid, Campus de Somosaguas, Madrid, Spain; ^4^Department of Psychology, New York University, New York City, NY, United States; ^5^Social Psychology and Motivation Division, Department of Psychology, University of Konstanz, Konstanz, Germany; ^6^Department of Psychology, University of Hamburg, Hamburg, Germany

**Keywords:** anger, mental contrasting with implementation intentions, reappraisal, self-regulation, emotion regulation

## Abstract

As anger can lead to aggressive behavior aiming at intentionally hurting somebody, the prevention of its destructive consequences with effective emotion regulation strategies is crucial. Two studies tested the idea that mental contrasting with implementation intentions (MCII) interventions would be effective in down-regulating anger. In Study 1, participants who adopted the self-regulation strategy of MCII showed significantly less anger-related negative affect after the anger induction than participants in a control condition, with positive affect staying unaffected. Results from a second study with a control condition plus three self-regulation conditions – a reappraisal, a MCII, and a reappraisal + MCII condition – suggest that participants using MCII were effective in down-regulating anger, irrespective of whether it was supplemented by reappraisal or not. The present research contributes to emotion regulation research by introducing MCII as an effective strategy that can be tailored to satisfy individual emotion regulation demands, such as dealing with experienced anger.

## Introduction

Most of the literature on anger focuses on its problematic consequences. The experience of anger affects not only the individual (e.g., higher levels of stress and poorer health; Diong et al., 2005), but also the individual’s social environment (e.g., in terms of social functioning; Lench, 2004). Anger has also been reported to play an important role in the development, maintenance, and treatment of emotional disorders (Cassiello-Robbins and Barlow, 2016), and is among the most challenging emotions faced in psychotherapy (Norcross and Kobayashi, 1999). However, few studies have addressed anger regulation (Szasz et al., 2011). Although anger might also have sometimes adaptive functions (Ein-Dor and Hirschberger, 2018; e.g., expressing anger in response to negative evaluations; Celik et al., 2016), we focused in this paper on the negative consequences of anger and thus on those situations where one might want to down-regulate anger (e.g., because it might predispose an individual to verbal aggression).

Previous anger regulation approaches include strategies such as relaxation coping skills (Deffenbacher et al., 1986) or rumination and distraction (Rusting and Nolen-Hoeksema, 1998). In this latter study, Rusting and Nolen-Hoeksema (1998) found that participants in a rumination condition were angrier than those in a distraction condition (and, even more so, than a control condition; Rusting and Nolen-Hoeksema, 1998, Study 3). These results are also in line with research by Anestis et al. (2009), suggesting that anger rumination is associated to physical aggression, verbal aggression, and hostility.

As noted before, rumination can be regarded as a maladaptive form of self-focused thinking. Other emotion-regulation strategies, even though they have been regarded as being potentially adaptive, can also turn out to be maladaptive. Research by Thomsen et al. (2013) shows, for example, that a form of self-focused thought known as reflection can also be maladaptive, as it was associated with an increase in avoidance symptoms in colon-cancer patients.

In contrast, one emotion regulation strategy that has been shown to be effective in down-regulating emotions (meta-analysis by Webb et al., 2012a), and in particular anger (e.g., Memedovic et al., 2010), is reappraisal. Reappraisal is a well-established emotion regulation strategy which may be conceived as changing the meaning of potentially emotion-relevant stimuli (e.g., Gross, 2015). Indeed, reappraisal was found to be more effective in down-regulating anger at the subjective and behavioral level (i.e., persistence on a frustrating task) than acceptance and suppression (Szasz et al., 2011). Reappraisal, as compared to suppression, led to reduced positive and negative emotional experience (Kalokerinos et al., 2015), and lowered blood pressure (Memedovic et al., 2010). In neuroimaging research, reappraisal differed from rumination in terms of functional connectivity (Fabiansson et al., 2012) and in terms of self-reported emotion and physiological responding: participants who reappraised reported less anger, as well as decreased central and peripheral sympathetic activation (Ray et al., 2008). Thus, reappraisal not only lowered anger feelings, but also physiological activation after participants thought of events that made them angry.

Contrary to these findings, however, reappraisal has been reported to be equally effective or even less effective than other emotion regulation strategies. A study by Germain and Kangas (2015) found, for example, that both reappraisal and suppression were effective in down-regulating the affective and physiological experience of state-anger, as compared to an acceptance group. Recently, Offredi et al. (2016) compared reappraisal to distraction and observed that distraction was even more effective than reappraisal and rumination. Taken together, most studies reveal a significant influence of reappraisal on the down-regulation of anger, whereas other research points to a lack of differential effectiveness when it comes to comparing reappraisal to other emotion regulation strategies.

One way of overcoming the discrepancies observed in the effectiveness of some emotion regulation strategies is by automatically instigating the targeted emotion regulation response (Schweiger Gallo et al., 2009). This strategic automaticity can be promoted via the self-regulation strategy of mental contrasting with implementation intentions (MCII; Oettingen, 2012). In mental contrasting (e.g., Oettingen, 2000, 2012), people first identify and imagine a desired future (e.g., not feeling angry when my colleague ignores me) and subsequently identify and imagine the inner obstacle of the present reality that stands in the way of realizing the wished-for future (e.g., feeling upset about my colleague’s inconsiderate behavior). Importantly, the MCII instructions guide participants toward identifying a desired future that is feasible and an obstacle that is within the person (i.e., inner obstacles are potentially surmountable). This juxtaposition of thoughts and images about a desired future and the inner obstacle of the present reality leads people to notice that they haven’t achieved their wish yet and it allows them to identify and think about whether and how to overcome the obstacle. If the obstacle is surmountable (expectations of overcoming the detected internal obstacle are high), people vigorously pursue their desired future.

The processes underlying the effectiveness of mental contrasting relate to changes in implicit cognition, implicit motivation, and responses to negative feedbacks (summary by Oettingen and Cachia, 2016). Specifically, mental contrasting leads to a change in the interpretation of the meaning of the present reality as an obstacle to the desired future (Kappes et al., 2013) and modulates the non-conscious associative link between the desired future and the obstacle, as well as the associative link between the obstacle and the instrumental behavior to surmount the obstacle (Kappes et al., 2012b; Kappes and Oettingen, 2014).

Next to changes in non-conscious cognition, mental contrasting of feasible wishes also motivates behavior by leading to heightened energization (measured by systolic blood pressure or feelings of energy) directed at overcoming the obstacle that stands in the way of the desired future and exerts its effectiveness via processes related to responding to setbacks (i.e., learning from negative feedback). At the same time, it lowers defensiveness to such set-backs (Kappes et al., 2012a). The described changes in non-conscious processes instigated by mental contrasting (i.e., pertaining to cognition, energization, and processing of set-backs) are found to mediate the subsequent actual behavior change.

When obstacles are particularly difficult to overcome, for example, when there are strong impulses or ingrained habits, planning how to overcome the obstacles is particularly relevant. Therefore, mental contrasting has been combined with the self-regulation strategy of implementation intentions. Implementation intentions come in the form of “If… (situation X), then I will… (show goal-directed behavior Y)!” They link the critical situation X to a specified goal-directed response Y (Gollwitzer, 1999). Implementation intentions can be used to strengthen the implicit associative link between obstacle and behavior to overcome the obstacle even further, because implementation intentions have been found to form a strong associative link between a critical situation (e.g., the inner obstacle) and the initiation of an instrumental response achieving the desired future. To explicate, by specifying implementation intentions in the context of mental contrasting the if-then plan can be formulated as: “If… (obstacle), then I will… (show behavior to overcome obstacle)!” In other words, the situation X refers to the person’s inner obstacle, and the goal-directed response refers to the behavior to overcome the obstacle (e.g., If I feel upset about my colleague, then I will consider how stressed out he is at the moment!; Oettingen, 2012). These goal-directed responses can target the prevention of unpleasant emotional responses (e.g., feeling anxious) elicited by avoidance-oriented emotions, but also of undesired behaviors (e.g., aggressive behaviors) associated with approach-oriented emotions such as anger (Carver and Harmon-Jones, 2009). If-then plans facilitate goal attainment due to the heightened activation and accessibility of the mental representation of the critical situation (e.g., Parks-Stamm et al., 2007) and a strong cue-response link (overview by Oettingen and Gollwitzer, 2015). Moreover, the strong associative link established between the critical situation and the goal-directed response leads to the automatic elicitation of the linked response when facing the critical situation (e.g., Bayer et al., 2009; Schweiger Gallo et al., 2009). Importantly, implementation intentions have been reported to be effective in the health domain and are indeed one of the most used strategies in health-behavior interventions (Hagger and Luszczynska, 2014).

The combination of mental contrasting with implementation intentions has been shown to be more effective than either strategy alone in promoting desired behavior such as becoming more cooperative (e.g., Kirk et al., 2013), as well as in preventing undesired behaviors such as reducing unhealthy eating habits (Adriaanse et al., 2010). MCII has also been shown to be effective in initiating and promoting goal-directed behavior when it comes to emotion regulation (e.g., reducing anxiety-based behaviors in romantic relationships; Houssais et al., 2013, and attenuating idiosyncratic anxieties; Brodersen and Oettingen, 2017). The regulation of emotions by implementation intentions has also received attention in research (overview by Webb et al., 2012b). For instance, implementation intentions were effective in regulating emotions such as sadness (Hallam et al., 2015) and, most recently, *grima* (i.e., the aversive experience to high-pitched, shrill sounds like the noise of a fork scratching a plate; Schweiger Gallo et al., 2017). Schweiger Gallo et al. (2009) have shown that implementation intentions convert even response-focused emotion regulation strategies into effective strategies. Indeed, both antecedent- and response-focused emotion regulation strategies proved effective when it came to the down-regulation of fear.

To date, however, little research has addressed anger regulation by implementation intentions (Rochat et al., 2016). In their case study, a patient trained to use implementation intentions to initiate previously established assertive behaviors successfully reduced the frequency and intensity of his anger outbursts.

In sum, MCII is a conscious self-regulation strategy which triggers automatic processes, that is, processes that efficiently run off outside of people’s awareness (Oettingen and Gollwitzer, 2015) which then predict behavior regulation. In other words, MCII profits from the automatic processes underlying the effectiveness of both mental contrasting and implementation intentions.

### The Present Research

Given that anger can cause impairments in the social, occupational, and romantic relationship domain (Lench, 2004), exploring the effectiveness of self-regulation strategies that target anger regulation seems vital. However, as Memedovic et al. (2010) noted, “there is a lack of empirically rigorous, systematic research on the regulation of anger” (p. 540). Though research on emotion regulation in general has experienced an exponential growth after the mid-1990s (Gross, 2015), further ways of effective emotion regulation are still to be discovered. This is all the more necessary for the regulation of anger, as such regulation is difficult to achieve (Mauss et al., 2007a).

In the present research we analyzed the effects of MCII on the regulation of anger by first examining whether an MCII intervention reduces anger feelings (Study 1). We expected that participants in the MCII condition would be more effective in down-regulating their anger feelings than those in a free recall control condition. Because we used a no treatment control condition in the first study, in Study 2 we went a step further and compared the anger regulation intervention based on MCII with an active intervention condition based on reappraisal, as well as a combined reappraisal + MCII intervention condition.

Given the findings regarding the effectiveness of reappraisal, on the one hand, and of MCII, on the other hand, we hypothesized that both MCII and reappraisal would be effective in downregulating anger, whereas the combined reappraisal + MCII strategy was expected to be even more effective. Thus we expected that participants engaged in reappraisal + MCII would outperform anger regulation as compared to reappraisal alone as well as MCII alone.

## Study 1: Mcii Vs. No-Treatment Control

Anger can be elicited by internal (e.g., feeling ashamed), external (e.g., the provocative behavior of others), and a combination of external stimuli and anger-related memories and images (Deffenbacher, 2011), such as overreacting to a touch because of the memories of being slammed in the face in the past. In fact, in a study by Glynn et al. (2007), the recall of a stressor was associated with increased blood pressure after 30 min and even after a week. Thus, in line with other research on anger regulation, where autobiographical recall of an event proved effective in inducing anger (e.g., Ray et al., 2008; Harmon-Jones et al., 2017) and even more effective than music and guided imagery (Jallais and Gilet, 2010), we asked participants to directly generate negative autobiographical events. This procedure was expected to prompt high feelings of anger related to a key experience in participants’ life.

### Method

#### Participants

Forty students at the University of Konstanz in Germany (mean age: 25.08 years, *SD* = 6.92; 77% females) participated in exchange for class credits or a financial bonus (5 Euro). A *post hoc* power analysis revealed that this sample granted 70% power to detect a medium-to-large effect size. The experiment adopted a 2-between (Condition: control, MCII) × 2-within (Time: baseline, post induction) × 3-within (Affect: anger, other negative, positive) mixed-factorial design. The participants were randomly assigned to either the control or MCII condition (20 participants per condition). The experiment was performed in accordance with the ethical standards laid down in the 1964 Declaration of Helsinki. In agreement with the ethics and safety guidelines at the University of Konstanz, we obtained a written informed consent statement from all individuals prior to their participation in the study. Potential participants were informed of their right to abstain from participation in the study or to withdraw consent to participate at any time.

#### Materials and Procedure

Participants first gave informed consent and then completed the 20 items of the German version (Breyer and Bluemke, 2016) of the PANAS by Watson et al. (1988) on a continuous scale as a baseline measure (**Table 1**). The PANAS comprises three items specifically associated with anger (i.e., upset, hostile, irritable), seven items associated with other negative affect, and ten items associated with positive affect. Next, participants completed measures on general self-efficacy (Schwarzer and Jerusalem, 1999) and subjective well-being (Dalbert, 1992). Because it has been suggested that people high in self-efficacy and optimism become angry less often (Ausbrooks et al., 1995), we wanted to control for these variables between participants in the control and self-regulation conditions at baseline.

**Table 1:** Overview of the procedure and the self-regulation instructions in Studies 1 and 2.

Measure/Manipulation	Description
1. Affect (baseline)	PANAS (3 anger items, 7 other negative affect items, 10 positive affect items)
2. Control variables	Self-efficacy (10 items), subjective well-being (6 items)
3. Anger induction	“Recall the worst interpersonal experience/event in the last 3 years that made you experience strong feelings of anger.”
4. *Study 2:* Reappraisal	[“Think about this interpersonal experience/event. How could you deal best with your anger? Please try to tell yourself that it would be preferable that the others are nice and/or fair to you, but if they are not, it does not mean that you or they are worthless human beings. It would be preferable that the others be nice and/or fair to you, but if they are not, remember that it is only (very) bad, not catastrophic (the worst thing that could happen to you). It would be preferable that the others would be nice and/or fair to you, but if they are not, you can tolerate it, and go on enjoying life, even if it‘s more difficult in the beginning.”]
5. Mental contrasting with implementation intentions (MCII)	“Think about this interpersonal experience/event. How could you deal best with your anger? [*Study 1:* Your wish is to deal competently with your anger feelings.] [*Study 2:* Take a deep breath and make yourself comfortable. It is important that you are not disturbed during the exercise. Start it when you feel calm and relaxed. And now imagine you could deal competently with your anger…] [*Study 1:* What would be the best, the best outcome, of fulfilling your wish?] [*Study 2:* What would be the best outcome of dealing competently with your anger?] How would you feel? The best outcome: _____ Now take a moment and imagine this outcome vividly and in detail. Write down your thoughts and ideas. Continue when you have a clear image of your best outcome. Sometimes things do not work as we want. [*Study 1:* Which is the main obstacle that prevents you from fulfilling your wish? What is it in you that hinders you from fulfilling your wish] [*Study 2:* What is it in you that prevents you from dealing competently with your anger? What is it in you that hinders you?] Please think twice. Is this the real, inner personal obstacle? Find out what really hinders you! Dig deeper into it! Find your inner obstacle. My inner obstacle: _____ Take a moment and imagine this inner obstacle vividly and in detail. Please write down your thoughts. Continue when you have a clear picture of your inner obstacle. [*Study 1:* What can you do to overcome the obstacle? Name an action that you can perform. Find your own action!] [*Study 2:* What can you do to overcome the obstacle? What could be an effective action or an effective thought to overcome the obstacle? Specify this action or thought and write it down. My action or thought: _____ ] Now make a plan: If … (obstacle occurs), then I will … (perform action). If: _____ then I will: ____ Read your if-then plan once more and imagine it!
6. Affect (post induction)	PANAS (3 anger items, 7 other negative affect items, 10 positive affect items)
7. Anger description	Written description of the anger event [*Study* *2*: ratings of intensity (How intense was the event?) on a 1 (not at all) to 100 (very) scale, recency (“How long ago is the event?”) on a 1 (not long) to 100 (very long) scale, and ease of recall (“How easy was it for you to recall the event?”) on a 1 (very easy) to 100 (very difficult) scale]
8. Final questionnaire	Demographics; [*Study 2*: demand to restrain anger (“Did you try hard to restrain your anger?”), to demonstrate restraint (“Did you want to show how good you can suppress your anger?”), and perceived request to restrain anger (“Did you remember the instruction to restrain anger?”) on 1 (does not apply) to 100 (applies) scales]


*The reappraisal instruction was presented in the reappraisal and the reappraisal + MCII conditions in Study 2. The MCII instruction was presented in the MCII condition in Studies 1 and 2 and in the reappraisal + MCII condition in Study 2.*

After these baseline measures, anger was induced by asking all participants to recall a relevant personal event. They then either received no emotion regulation strategy (control condition) or were guided through the MCII procedure (MCII condition): Participants in the MCII condition were first asked to generate the wish of dealing competently with their anger feelings. Next, they identified and imagined the best outcome that would result from fulfilling their wish before they identified and imagined the internal obstacle that might prevent them from fulfilling their wish. In a last step, participants in the MCII condition formed an “if… *personal obstacle*, then I will… *response* *to overcome obstacle*” plan. Finally, participants in both the control and the MCII condition again filled out the PANAS and then wrote about their anger evoking event. All materials were administered in paper-and-pencil format. Finally, they were thoroughly debriefed.

### Results and Discussion

Conditions did not differ on baseline measures of self-efficacy or subjective well-being, *ps* > 0.3. We subjected affective ratings to a 2-between (Condition: control, MCII) × 2-within (Time: baseline, post induction) × 3-within (Affect: other negative, positive, anger) mixed ANOVA. A main effect of time, *F*(1, 38) = 79.75, *p* < 0.001, $\eta_{p}^{2}$ = 0.68, 90% CI [0.52, 0.76], was governed by an interaction of time and affect, *F*(1.6, 61.5) = 78.96, *p* < 0.001, $\eta_{p}^{2}$ = 0.68, 90% CI [0.55, 0.74], reflecting a strong increase of anger, *t*(112.8) = 13.73, *p* < 0.001, *d* = 2.17, 95% CI [1.59, 2.74], a smaller increase in other negative affect, *t*(112.8) = 6.17, *p* < 0.001, *d* = 0.98, 95% CI [0.59, 1.35], and a decrease in positive affect, *t*(112.8) = 3.36, *p* = 0.003, *d* = 0.53, 95% CI [0.20, 0.86], thus indicating that the recall task successfully induced anger.

**FIGURE 1:**
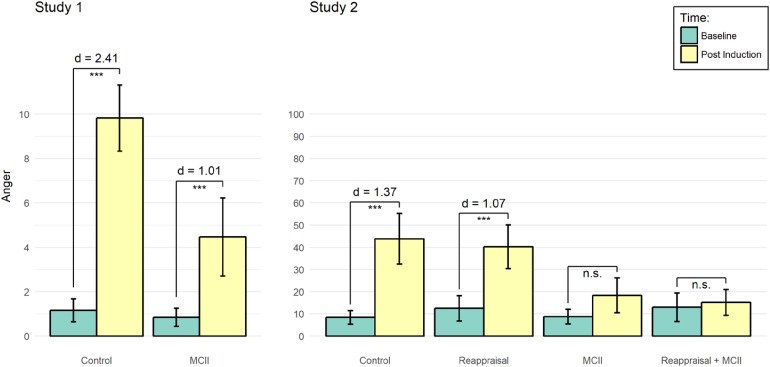
Mean ratings of reported anger by conditions at baseline and post induction, separately for Studies 1 and 2. Error bars represent 95% confidence intervals. ^∗∗∗^*p* < 0.01.

Further, the three-way interaction, *F*(1.6, 61.5) = 9.26, *p* < 0.001, $\eta_{p}^{2}$ = 0.20, 90% CI [0.06, 0.32], suggests that this pattern differed between conditions. A separate Condition × Time ANOVA for positive affect only revealed a main effect of time, *F*(1, 38) = 26.12, *p* < 0.001, $\eta_{p}^{2}$ = 0.41, 90% CI [0.20, 0.55], reflecting that participants reported more positive affect at baseline than post induction. However, for negative affect and anger the interaction effect of Condition and Time was significant, *F*(1, 38) = 5.97, *p* = 0.019, $\eta_{p}^{2}$ = 0.14, 90% CI [0.01, 0.30] and *F*(1, 38) = 19.62, *p* < 0.001, $\eta_{p}^{2}$ = 0.34, 90% CI [0.14, 0.49], respectively. Participants in the control condition reported a strong increase in negative affect, *t*(38) = 6.20, *p* < 0.001, *d* = 1.39, 95% CI [0.76, 2.00], while a weaker effect evinced in the MCII condition, *t*(38) = 2.74, *p* = 0.019, *d* = 0.61, 95% CI [0.13, 1.08]. Similarly, participants in the control condition reported a very strong increase in anger, *t*(38) = 10.77, *p* < 0.001, *d* = 2.41, 95% CI [1.52, 3.28], while a weaker effect evinced in the MCII condition, *t*(38) = 4.51, *p* < 0.001, *d* = 1.01, 95% CI [0.46, 1.54]. As predicted, only MCII participants were able to down-regulate anger (**Table 2** and **Figure 1**). In Study 2, we went one step further by opting for an active treatment control condition: reappraisal.

**Table 2:** Means (standard deviations) of affective ratings in Study 1.

	Baseline			Post induction		
	Anger	Negative	Positive	Anger	Negative	Positive
Control	1.2 (1.1)	2.3 (1.6)	7.4 (1.1)	9.8 (3.2)	6.1 (2.9)	5.8 (1.7)
MCII	0.8 (0.9)	1.9 (1.2)	8.0 (1.9)	4.5 (3.8)	3.6 (2.5)	6.6 (1.9)


## Study 2: Mcii Vs. Reappraisal

### Method

#### Participants

One-hundred and thirty-seven German students at the University of Konstanz (females 99; mean age *M* = 22.15, *SD* = 5.09) completed the study in exchange for class credits or a financial bonus (5 Euro). Three participants could not complete the study due to technical problems. Participants gave informed consent and were randomly assigned to a control or to one of three self-regulation conditions (i.e., reappraisal, MCII, or reappraisal + MCII), with 33, 36, 33, and 32 participants per condition, respectively. At the end of the study, participants were thoroughly debriefed. Power for detecting medium-to-large-sized effects in this analysis was high (98%). The experiment adopted a 4-between (Condition: control, reappraisal, MCII, reappraisal + MCII) × 2-within (Time: baseline, post induction) × 3-within (Affect: anger, other negative, positive) mixed-factorial design. The experiment was performed in accordance with the ethical standards laid down in the 1964 Declaration of Helsinki. In agreement with the ethics and safety guidelines at the University of Konstanz, we obtained a written informed consent statement from all individuals prior to their participation in the study. Potential participants were informed of their right to abstain from participation in the study or to withdraw consent to participate at any time.

#### Materials and Procedure

The procedure was the same as in Study 1, except that all materials were delivered at the computer and answers were provided on 1 to 100 visual analog scales. The computerized assessment allowed us to determine how long participants worked on their respective instructions. Reappraisal and reappraisal + MCII condition participants received the reappraisal instructions taken from Szasz et al. (2011). At the end of the study, we added questionnaires to address potential alternative explanations of the expected findings. As differences in the characteristics of the anger-evoking event might explain potential condition differences in affective ratings, we assessed the intensity (“How intense was the event?”), recency (“How long ago is the event?”), and ease of recall (“How easy was it for you to recall the event?”) of the event with scales ranging from 1 (not at all/not long/very easy) to 100 (very/very long/very difficult), respectively.

Further, the self-regulation instructions in the different conditions might have been differentially perceived as a demand to restrain one’s anger. To account for this latter possibility, we measured how strongly participants tried to restrain their anger (“Did you try hard to restrain your anger?”), to demonstrate successful restraint (“Did you want to show how good you can restrain your anger?”), and whether they perceived the instruction as being a request to restrain their anger (“Did you remember the instruction to restrain anger?”). All questions were answered on visual analog scales ranging from 1 (does not apply) to 100 (applies).

### Results and Discussion

Conditions did not differ with respect to subjective self-efficacy or well-being, *p*s > 0.10. We subjected affective ratings to a 4-between (Condition: control, reappraisal, MCII, reappraisal + MCII) × 2-within (Time: baseline, post induction) × 3-within (Affect: other negative, positive, anger) mixed ANOVA. A main effect of time, *F*(1, 130) = 46.55, *p* < 0.001, $\eta_{p}^{2}$ = 0.26, 90% CI [0.16, 0.36], was governed by an interaction of time and affect, *F*(1.6, 207.6) = 43.50, *p* < 0.001, $\eta_{p}^{2}$ = 0.25, 90% CI [0.17, 0.33], reflecting a strong increase of anger, *t*(383.9) = 10.75, *p* < 0.001, *d* = 0.93, 95% CI [0.72, 1.13], a smaller but significant increase in other negative affect, *t*(383.9) = 3.90, *p* < 0.001, *d* = 0.34, 95% CI [0.16, 0.51], and no changes in positive affect, *p* = 0.207. This finding demonstrates that the recall task successfully induced anger.

The three-way interaction effect was significant as well, *F*(4.8, 207.6) = 7.53, *p* < 0.001, $\eta_{p}^{2}$ = 0.15, 90% CI [0.07, 0.21], suggesting that conditions differed in their affective ratings. This pattern of results held also true when adjusting for a possible confounding effect of instruction length (i.e., the potentially longer time devoted to processing the instructions by the self-regulation condition participants), *F*(4.7, 206.0) = 2.75, *p* = 0.022, $\eta_{p}^{2}$ = 0.06, 90% CI [0.01, 0.10]. The instruction length itself had no significant main effect and did not interact with any other variable, *Fs* < 1, *ns*. These results indicate that any differences between conditions with regard to their affective ratings cannot be attributed to differences in the length of the instructions given. To follow up the three-way interaction effect, we conducted separate Condition × Time ANOVAs for positive affect, negative affect, and anger. Regarding positive affect, only a main effect of time evinced, *F*(1, 130) = 7.03, *p* = 0.009, $\eta_{p}^{2}$ = 0.05, 90% CI [0.01, 0.12], reflecting that participants reported more positive affect at baseline than post induction. Regarding negative affect as well as anger, we observed interaction effects of Condition and Time, *F*(3, 130) = 5.62, *p* = 0.001, $\eta_{p}^{2}$ = 0.12, 90% CI [0.03, 0.19], and *F*(3, 130) = 11.84, *p* < 0.001, $\eta_{p}^{2}$ = 0.21, 90% CI [0.11, 0.30], respectively. Participants in the control condition, *t*(130) = 4.54, *p* < 0.001, *d* = 0.79, 95% CI [0.39, 1.18], and the reappraisal condition, *t*(130) = 3.76, *p* = 0.001, *d* = 0.63, 95% CI [0.26, 0.98], reported more negative affect post induction than at baseline. No such differences between baseline assessments and post induction measures were found in the MCII and the MCII + reappraisal conditions. Similarly, participants in the control condition, *t*(130) = 7.89, *p* < 0.001, *d* = 1.37, 95% CI [0.89, 1.85], and the reappraisal condition, *t*(130) = 6.45, *p* < 0.001, *d* = 1.07, 95% CI [0.66, 1.48], reported more anger post induction assessments than at baseline. Again, no such differences were found in the MCII and the MCII + reappraisal conditions. Corroborating the results of Study 1, only participants with a MCII strategy were successful in down-regulating their experienced anger (**Table 3** and **Figure 1**).

**Table 3:** Means (standard deviations) of affective ratings in Study 2.

	Baseline			Post induction		
	Anger	Negative	Positive	Anger	Negative	Positive
Control	8.4 (8.6)	12.5 (10.9)	49.2 (14.0)	43.8 (32.1)	27.5 (24.6)	43.7 (13.0)
Reappraisal	12.5 (16.9)	16.7 (13.5)	48.4 (15.0)	40.2 (29.2)	28.6 (21.2)	42.8 (15.7)
MCII	8.7 (9.2)	16.1 (14.5)	51.6 (15.9)	18.3 (22.1)	15.4 (13.5)	52.1 (21.1)
Reappraisal + MCII	12.9 (17.7)	14.4 (11.6)	48.6 (16.4)	15.1 (16.1)	15.5 (11.9)	46.5 (18.6)


Regarding potential alternative explanations of these findings, (marginally) significant differences between conditions emerged regarding anger intensity, the demand to demonstrate restraint, and the perceived request to restrain anger, *p*s < 0.08. Specifically, reappraisal participants reported marginally more intensity of their anger experience than MCII participants, *t*(130) = 2.59, *p* = 0.065, *d* = 0.63, 95% CI [0.13, 1.12], and significantly less effort to demonstrate restraint than reappraisal + MCII participants, *t*(98) = 2.49, *p* = 0.043, *d* = 0.61, 95% CI [0.10, 1.11]. Moreover, reappraisal + MCII participants tended to perceive their instruction more strongly as a demand to restrain anger than reappraisal participants, *t*(98) = 2.19, *p* = 0.092, *d* = 0.54, 95% CI [0.04, 1.03], and MCII participants, *t*(98) = 2.40, *p* = 0.054, *d* = 0.60, 95% CI [0.09, 1.09]. Importantly, however, adding these variables as covariates to the analysis of affective ratings did not change our results, ruling out differences regarding the anger experience and demand characteristics as alternative explanations of our findings.

## General Discussion

Across two studies, we found that MCII, a self-regulation strategy of goal pursuit, enabled participants to effectively reduce their anger. Participants who underwent an MCII intervention were able to successfully down-regulate their anger feelings as compared to participants in a control condition (Study 1). In a second study, we compared anger regulation by MCII to anger regulation via reappraisal, as well as a combined reappraisal + MCII strategy. Results revealed that participants using MCII were effective in down-regulating anger, irrespective of whether it was supplemented by reappraisal or not. Thus, the present studies confirm the effectiveness of MCII, and they extend previous findings where MCII was shown to attenuate problems in samples with impairments in emotion regulation, such as in depressed patients (Fritzsche et al., 2016) and in children with symptoms of ADHD (Gawrilow et al., 2013) to the domain of emotion regulation and specifically anger regulation.

MCII is a meta-cognitive strategy enhancing self-regulation via automatic processes that can be adapted to one’s own emotion regulation needs. Indeed, the MCII procedure requires a person to consciously engage in juxtaposing a desired future with the inner obstacle preventing them from attaining their future, as well as to specify how they want to overcome the obstacle before they make a respective if-then plan. Though the mental juxtaposition of the desired future and the obstacle, as well as the formation of the if-then plan are conscious processes, once individuals have engaged in MCII, the cognitive and motivational processes mediating behavior change run off non-consciously, that is, without that people are aware of them. Thus people can automate their emotion regulation by filling into the MCII exercise the specific contents that they feel will help them balance their emotions during everyday life. Indeed, MCII interventions allow individuals to take advantage of their idiosyncratic, personalized experiences instead of relying on outside (i.e., the therapists or the researcher’s) standardized advice on how to down-regulate their anger. They can target their personal wishes and internal obstacles (e.g., intrusive thoughts, heightened arousal) and create the plans that fit best to their strengths and vulnerabilities of how to deal with these obstacles (e.g., relaxation strategies, behavioral strategies, cognitive strategies or any combination of them; Deffenbacher, 2011). Whereas some individuals might opt to keep calm and relaxed when encountering an anger-evoking stimulus, others might target changing their anger-eliciting thoughts or choose to focus on their conflict-management skills. Further, the framing of the planned responses is variable (e.g., approaching or avoiding specific anger states). Thus, MCII allows people to focus on their preferred way of regulating their emotions. Whether people choose to distract themselves, reappraise, or keep calm, MCII should aid in automating these ways of responding to their emotions. In all, the MCII exercise can be tailored to satisfy specific necessities of the situation the person finds herself in (e.g., adjust cognitions; modify bodily, facial, verbal, or behavioral responses).

In his prominent process model of emotion regulation, Gross (1998) distinguishes between antecedent- and response-focused emotion regulation strategies. Whereas antecedent-focused emotion regulation strategies unfold their effects before emotion responses are even generated, response-focused emotion regulation strategies work by modifying emotional responses once they have been generated. Examples of antecedent-focused emotion regulation strategies include situation selection, situation modification, and reappraisal, whereas suppression is regarded as a response-focused emotion regulation strategy because it aims at changing emotion expressions. One fundamental advantage of using MCII is that both antecedent-focused or response-focused emotion regulation strategies can be incorporated into the then-part of the if-then plans. Thus, one person may prefer to specify distracting oneself when anticipating their inner obstacle to successful anger regulation, whereas another person might specify changing facial expressions. Further, even emotional reactions to experienced emotions can be targeted (Yih et al., 2018) in the if-then plan (e.g., when feeling envy makes one feel anger). In sum, regardless of the chosen emotion regulation plans, MCII should increase the effectiveness of emotion regulation strategies such as response-focused strategies due to the highly idiosyncratic and automatic elicitation of the specified responses.

### Limitations

Our studies share methodological limitations with other anger studies, including the use of non-clinical populations such as college students and the reliance on self-reported data. Though relying on young adults may be seen as a limitation, the fact that the effectiveness of MCII has been shown in populations other than convenience samples and across the academic, interpersonal, and health domain (Oettingen et al., 2015) weakens this objection. In addition, as younger adults have been found to report more anger than older adults (e.g., Blanchard-Fields and Coats, 2008), relying on a college population seems justified.

Another possible criticism relates to the induction of anger. First, the levels of experienced anger were generally higher in Study 1 than in Study 2 although we used the same induction method in both cases. This ceiling effect might explain why MCII participants in Study 1 but not in Study 2 displayed significant increases in anger from baseline to post induction (**Figure 1**). The differences in experienced total anger between Study 1 and Study 2 might also reflect differences in how people used the different scales (while Study 1 was a paper-and-pencil assessment, Study 2 was a computerized assessment) or might have resulted from variations in the experiences participants recalled in the two studies. Second, we cannot rule out an inherent anger regulation by our participants (see Gilam and Hendler, 2015), meaning that they down-regulated their anger experience after the induction irrespective of the intervention. Importantly, however, possible inherent regulation should have influenced anger regulation in all experimental conditions alike.

Though we followed previous research, in which anger was induced before participants were assigned to their self-regulation conditions (e.g., Szasz et al., 2011), our results should still be interpreted with caution, as we did not compare the effects of the different instructions to a baseline measure before the anger induction. We chose this induction method because we suspected that giving participants a self-regulation strategy right from the beginning might have affected their anger experience at baseline. However, this in turn implies that the anger induction method might have impacted the anger ratings. Thus, future research might want to complement the procedure of the present research by using other anger induction methods (e.g., interpersonal anger provocations; Gilam and Hendler, 2015), as well as other study designs (e.g., Fabiansson et al., 2012).

Finally, the decrease in other negative emotions also deserves mention. Given that participants engaging in the MCII procedure are not provided with instructions specifying how to deal with their anger feelings, but rather identify their personal obstacles and form their own, personalized plans, it may be the case that their plans targeted the regulation of anger in a way that other negative emotions were also downregulated. Indirect support for this assumption is provided by research on emotion regulation with experimenter-provided implementation intentions, in which no such generalization effects were observed (Schweiger Gallo et al., 2017): implementation intention participants who formed the plan “And if I hear a *grima*-eliciting sound, then I will ignore it!” managed to down-regulate their *grima*-experience, but did not differ from goal intention participants with regards to their reactions to pleasant, unpleasant and disgust-eliciting sounds. Another explanation may be that the PANAS scale includes negative affect items which might have been linked to the anger experience, such as feeling distressed or nervous.

### Future Directions

MCII interventions may also be used to complement cognitive interventions, such as cognitive-behavioral therapies, which are among the most common anger treatment approaches (Beck and Fernandez, 1998). Because angry patients have been reported to believe that the behavior of other people is geared toward harming them (DiGiuseppe et al., 1994), and the effects of MCII are based on controlling automatic cognitions, interventions encompassing MCII should be well-suited to attenuate anger also in a clinical context (e.g., Sailer et al., 2015). Finally, because MCII rests on automatic processes, MCII interventions may attenuate the physiological and cognitive costs of anger experiences such as maladaptive cardiovascular responding (Mauss et al., 2007b) or impaired memory (Gross, 2015). As these are only speculations, however, future research should investigate these questions.

The lack of effectiveness of reappraisal deserves particular mention. One possible explanation has been provided by research underscoring the importance of moderating variables, such as conditions of high emotional intensity. Indeed, it has been suggested that reappraisal is less successful than distraction when it is initiated late in the information processing stream, as overriding already established thoughts gets more difficult (Sheppes and Meiran, 2007). Further, the combined reappraisal + MCII may have failed to outperform MCII in Study 2 due to a floor effect (i.e., MCII already down-regulated anger effectively). Given the limitations of the present research – including the failure to replicate previous findings indicating decreased anger following reappraisal, as well as the unspecific attenuation of both anger and other negative emotions caused by MCII – future research may complement the present findings with different anger induction methods and study designs to fill these gaps. Further, the combined reappraisal + MCII may have failed to outperform MCII due to a floor effect (i.e., MCII already down-regulated anger effectively).

## Summary

In two studies, we found an MCII intervention to be effective in down-regulating acutely induced anger. As compared to other anger treatment approaches, which may extend over several sessions (meta-analysis by Del Vecchio and O’Leary, 2004), MCII can be taught and applied in a cost- and time effective way. This being said our experiment was not set up to make claims about the effectiveness of MCII in comparison to other self-regulation strategies commonly used in emotion research. Future research should compare MCII to other strategies by applying different anger induction methods and study designs. Based on Denson et al. (2012) who highlight the potential role of increased self-control for the reduction of aggression, we hope that improving anger regulation via interventions such as MCII may ultimately lead to better individual and social functioning.

## Author Contributions

ISG, PG, and GO formulated the research question. ISG, MB, PG, and GO designed the studies. ISG, MB, and MA collected the data. ISG, MB, and MA analyzed the experimental results. ISG wrote the article and all authors were involved in editing and revising the early drafts and approving the final manuscript.

## Conflict of Interest Statement

The authors declare that the research was conducted in the absence of any commercial or financial relationships that could be construed as a potential conflict of interest.
